# The influence of socioeconomic aspects and hospital case volume on survival in colorectal cancer in Saxony, Germany

**DOI:** 10.1186/s12885-023-10672-1

**Published:** 2023-03-10

**Authors:** Andreas Bogner, Jürgen Weitz, Daniela Piontek

**Affiliations:** 1 Department of Visceral, Thoracic and Vascular Surgery, Faculty of Medicine Carl Gustav Carus, University Hospital, Technische Universität Dresden, Dresden, Germany; 2 Joint Office of the Clinical Cancer Registries in Saxony, The State Chamber of Physicians of Saxony, Dresden, Germany; 3 National Center for Tumor Diseases (NCT/UCC) & German Cancer Research Center (DKFZ) & University Hospital and Faculty of Medicine Carl Gustav Carus & Technische Universität Dresden & Helmholtz-Zentrum Dresden-Rossendorf (HZDR), Dresden and Heidelberg, Germany

**Keywords:** Colorectal cancer, Survival, Laparoscopic surgery, Hospital volume, German index of socioeconomic deprivation (GISD)

## Abstract

**Background:**

Colorectal cancer (CRC) is one of the most common types of cancer in Western civilization and responsible for a high number of yearly deaths. Long-term outcome is influenced by many factors, potentially including socioeconomic aspects like income, education, and employment. Furthermore, annual surgical case volume plays a major role in achieving good oncological results. In our retrospective study, we evaluated the effect of socioeconomic deprivation and hospital volume on overall survival (OS) in the federal state of Saxony, Germany.

**Methods:**

All patients with CRC who underwent surgery in Saxony, Germany between 2010 and 2020 and were living in Saxony at the time of diagnosis were included in our retrospective analysis. Uni- and multivariate analyses were conducted considering age, sex, tumor localization, UICC tumor stage, surgical approach (open/laparoscopic), number of resected lymph nodes, adjuvant chemotherapy, year of surgery, and hospital case volume. In addition, our model was adjusted for social disparity using the German Index of Socioeconomic Deprivation (GISD).

**Results:**

A total of 24,085 patients were analyzed (15,883 with colon cancer and 8,202 with rectal cancer). Age, sex, UICC tumor stage and tumor localization were distributed as expected for CRC. Median overall survival time was 87.9 months for colon cancer and 110.0 months for rectal cancer. Univariate analysis revealed laparoscopic surgery (colon and rectum *P* < 0.001), high case volume (rectum: *P* = 0.002) and low levels of socioeconomic deprivation (colon and rectum *P* < 0.001) to be significantly associated with better survival. In multivariate analyses, the associations of laparoscopic surgery (colon: HR = 0.76, *P* < 0.001; rectum: HR = 0.87, *P* < 0.01), and mid-low to mid-high socioeconomic deprivation (colon: HR = 1.18–1.22, *P* < 0.001; rectum: HR = 1.18–1.36, P < 0.001–0.01) remained statistically significant. Higher hospital case volume was associated with better survival only in rectal cancer (HR = 0.89; *P* < 0.01).

**Conclusion:**

In Saxony, Germany, better long-term survival after CRC surgery was associated with low socioeconomic deprivation, laparoscopic surgery and partly with high hospital case volume. Thus, there is a need to reduce social differences in access to high-quality treatment and prevention and increase hospital patient volume.

**Supplementary Information:**

The online version contains supplementary material available at 10.1186/s12885-023-10672-1.

## Background

Colorectal cancer (CRC) is the third most common cancer worldwide and the second leading cause of cancer-related deaths every year [1]. In Germany, CRC accounts for approximately 60,000 newly diagnosed tumors and around 25,000 tumor-related deaths annually. Due to the implementation of health programs offering colonoscopy as a widely available examination for early detection and screening, the incidence and mortality of CRC is only slowly declining [2]. Many diseases, including cancer, follow a socioeconomic gradient with higher rates in patients with lower socioeconomic status [3, 4]. In Germany, an aging society is being confronted with the risk of increasing social disparities. In view of the structure of the German healthcare system, which grants free access to healthcare for everyone, as well as several campaigns in the media, cancer therapy and outcome should theoretically be equally distributed. Since individual-level socioeconomic data are difficult to obtain, especially in large cohorts, the German Index of Socioeconomic Deprivation (GISD) can be used as an external indicator. It considers the dimensions of education, employment and income, with each contributing one-third to the overall score [5, 6]. Jansen et al. showed a worse relative survival of patients living in the most deprived districts compared to the survival of patients living in more privileged areas throughout Germany, regarding the 25 most common cancer sites in ten population-based registries (covering 32 million inhabitants).[7].

However, socioeconomic disparity is not the only factor influencing survival in CRC. High individual surgeon and institutional case volume is a well-documented factor influencing outcomes in oncological surgery. [8]. The centralization of care to “centers of excellence” in Europe has improved oncological outcomes over the last decades [9]. It has also been shown that treating patients in high-volume hospitals by high-volume surgeons is associated with better in-hospital mortality and oncological outcome [10, 11]. Even though centralization of cancer care is expected to yield superior results, the national strategy in Germany is still based on a voluntary certification process. Oncological colorectal surgery can still be performed in almost every hospital by every surgeon without restrictions [12, 13]. Nevertheless, only about one-third of CRC patients in Germany are treated in high-volume centers.

In our retrospective analysis, we wanted to examine socioeconomic disparities using the GISD, and the influence of annual hospital case volume for colorectal disease and their influence on survival in the federal state of Saxony, Germany.

## Methods

### Study population

For the present retrospective analyses, we used data from the four clinical cancer registries in Saxony, Germany. This study does not include data of a clinical trial. In Germany, all inpatient and outpatient physicians as well as pathologists are obliged to report information on diagnosis, histological results, treatment and outcomes to the clinical cancer registries. Patients need to be informed about this process. Consent is not required but, patients have a right of objection, which is hardly used (fewer than five cases/year in Saxony). After documentation and validation of the data, analyses are conducted with an anonymized data set that does not allow identification of individual patients. Completeness of the registries has been estimated to be 98% across all tumors since 2007 [14].

**Fig. 1:**
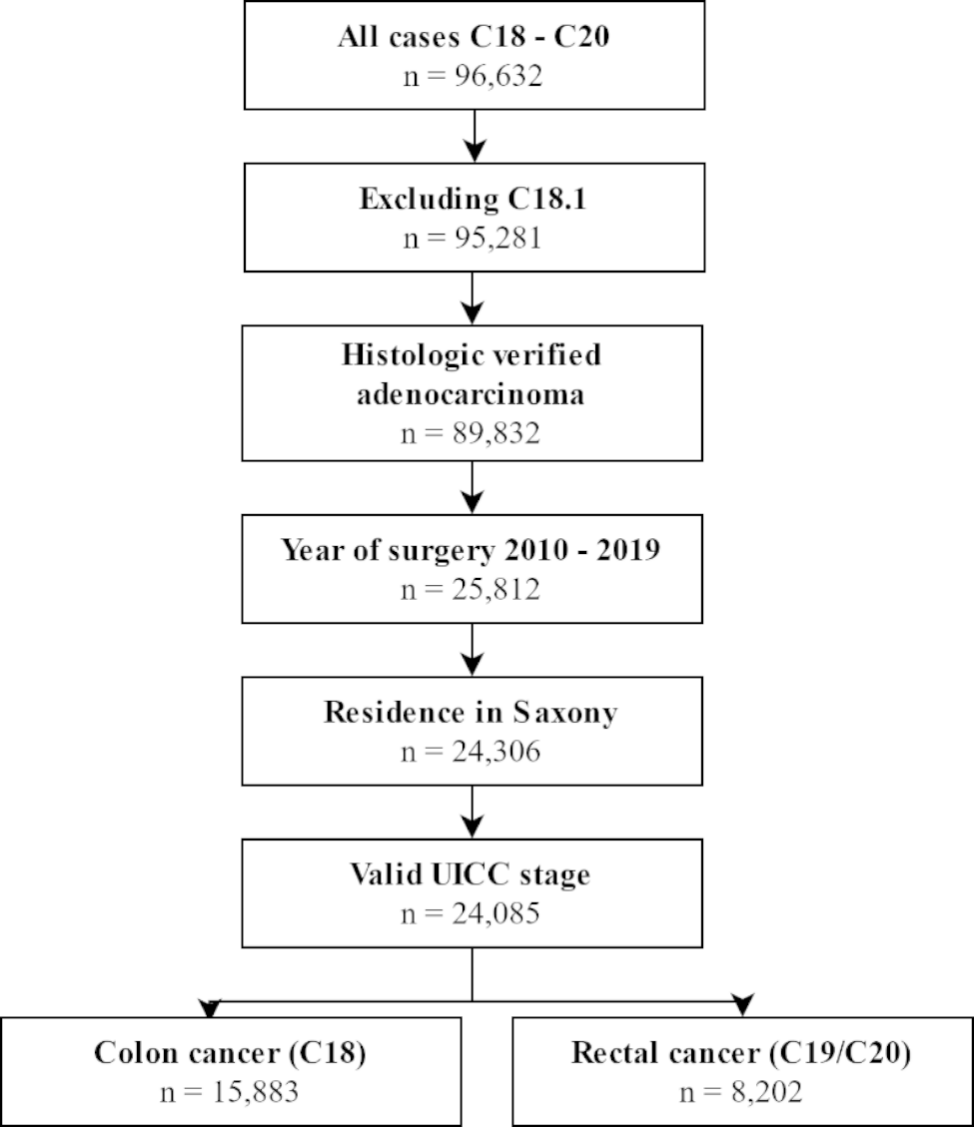
Flowchart of data collection, inclusion criteria and patient selection Abbreviations: UICC, Union for International Cancer Control.

All cases with ICD-10 diagnoses C18 (excluding C18.1), C19 and C20, histologically verified adenocarcinoma and tumor-specific surgery in Saxony in the years 2010 to 2020 were included (n = 25,812). Patients had to be at least 18 years old and have their main residence in Saxony at the time of diagnosis (n = 24,306). We excluded 221 cases with missing information on UICC tumor stage. Thus, the final analytical sample comprised 24,085 cases (Fig. 1).

#### Included variables and statistical analyses

**Fig. 2:**
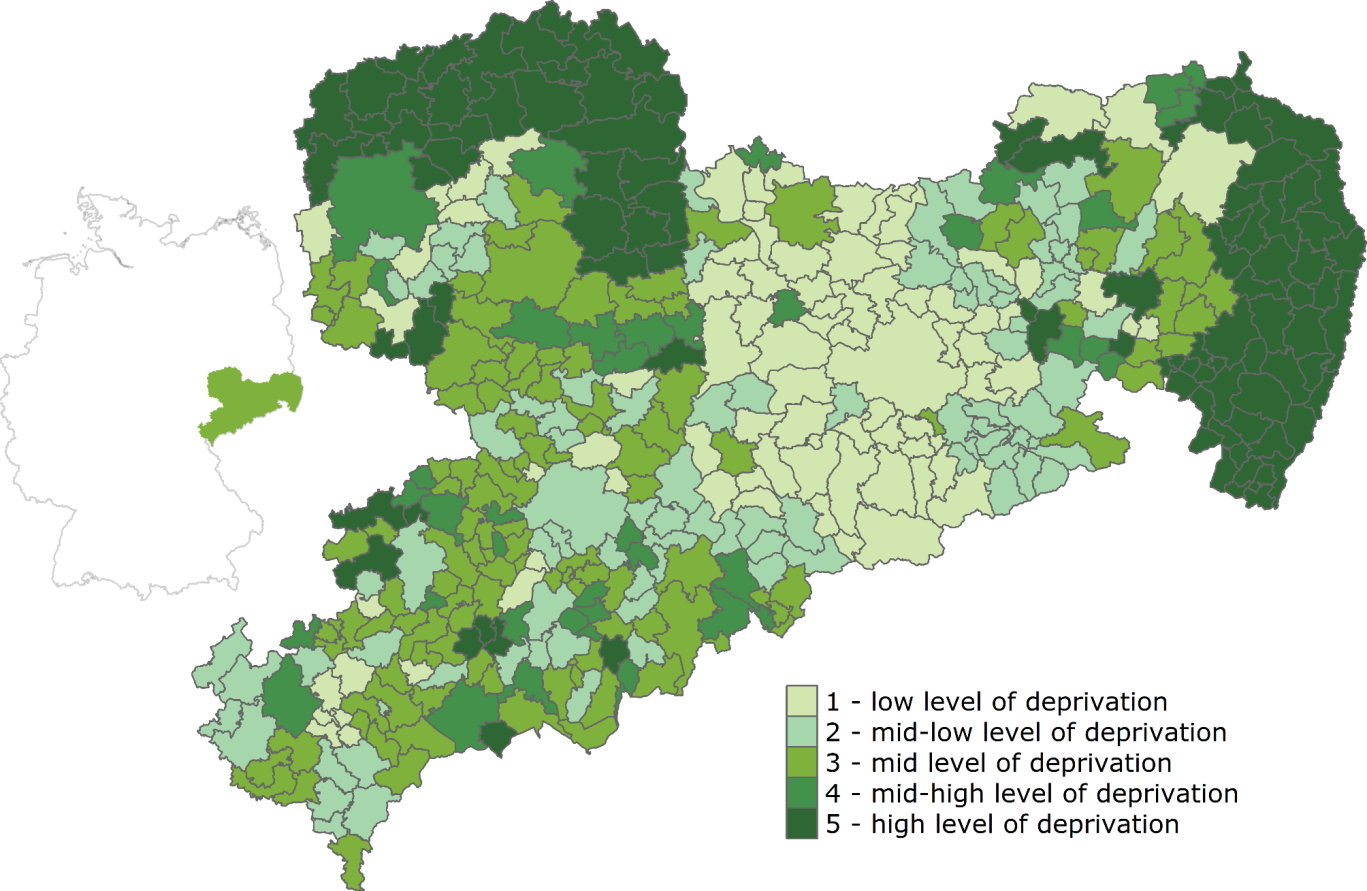
Distribution of German Index of Socioeconomic Deprivation (GISD) quintiles in Saxony, Germany

Tumor localization was documented according to the International Classification of Diseases for Oncology (ICD-O; C18.0, C18.2 to C18.9, C19.9, C20.9). For multivariate analyses, colon cancer was further classified into right-sided (C18.0, C18.2–C18.4), left-sided (C18.5–C18.7) and others (C18.8, C18.9). We considered tumor stages defined by the Union for International Cancer Control (UICC; stages I, II, III, IV). Post-surgical histopathological classification of the UICC was used [15]. Regarding surgical treatment, only tumor-specific resections were considered. In the case of more than one resection, only the first procedure was analysed. The surgical approach was classified into open surgery, laparoscopic surgery, conversion and others/not specified. The urgency of surgery was documented as elective surgery vs. emergency surgery vs. unknown/missing. The documented number of resected lymph nodes was dichotomized into less than 12 and at least 12 lymph nodes. We further dichotomized patients based on the annual case volume of the hospital which was divided into low vs. high using a cut-off of 20 and 30 surgeries per year for rectal cancer (C19/C20) and colon cancer (C18), respectively. Adjuvant chemotherapy was documented if it was explicitly stated as a treatment option or based on the interval between surgery and start of chemotherapy (maximum time difference: 20 weeks). As an additional external indicator, a measure of area-based socioeconomic deprivation at the level of municipalities was used. The German Index of Socioeconomic Deprivation (GISD) considers the dimensions of education, employment and income, with each contributing one-third to the overall score [6]. Higher values indicate higher deprivation, i.e., lower socioeconomic status. For each tumor case, the GISD score of the patient’s place of residence at the time of diagnosis was used. The municipalities were then grouped into GISD quintiles based on our final analytical sample (1 – low level of deprivation, 2 – mid-low level of deprivation, 3 – medium level of deprivation, 4 – mid-high level of deprivation, 5 – high level of deprivation; see Fig. 2).

For descriptive purposes, absolute numbers, and percentages (categorical variables) as well as medians (continuous variables) are presented. Absolute overall survival (OS) was calculated based on Kaplan–Meier estimation. Median survival time as well as 5-year survival rates with corresponding 95% confidence intervals are reported. Univariate survival analyses were conducted for hospital case volume, surgical approach, and socioeconomic deprivation. For descriptive comparisons, the survival time of different groups of these categorical variables was plotted as Kaplan–Meier curves. Log-rank tests were used to test whether survival time differed between the included groups. Multivariate analyses were performed using weighted Cox regression, which provides unbiased average hazard ratio estimates even in the case of non-proportional hazards [16]. The resulting hazard ratios (HR) can be interpreted as relative risks. Survival analyses included only primary tumors and cases with a minimum follow-up time of one month (n = 19,845). Statistical analyses and graphical illustration were carried out using the R software package (version 3.6.0, https://www.R-project.org, The R Foundation).

## Results

### Patient characteristics

A total of 24,085 patients were included in the analysis (15,882 with colon cancer and 8,202 with rectal cancer). The description of the included colon and rectum cancer cases is shown in Table 1. The sample was predominantly male, also in terms of the proportion of cases of rectal cancer compared to colon cancer (64.8% vs. 54.6%). The median age was 70 and 74 years, respectively. The majority of colon cancer cases was localized on the right-side (58.9%). Metastatic tumor stage (UICC IV) was present in 17.3% (colon) and 13.9% (rectum) of all cases. In most surgeries, at least 12 lymph nodes were resected (colon: 92.5%, rectum: 87.6%). The proportion of surgeries performed using a laparoscopic approach was higher for rectal cancer than for colon cancer (24.8% vs. 14.8%). Emergency surgery was more prevalent in colon cancer than in rectal cancer (7.5% vs. 1.8%). High hospital case volume was found in 56.1% of colon cancer resections and 45.3% of rectal cancer resections. Socioeconomic deprivation was evenly distributed due to the use of quintiles. The distribution of patient characteristics by case volume is shown in Supplementary Tables 1 and 2.

Colon resections were conducted in a total of 64 hospitals. There were 43 low-volume hospitals with a median number of 16.7 surgeries per year (minimum 0.1, maximum 29.1) and 21 high-volume hospitals with a median number of 42.7 surgeries per year (minimum 30.0, maximum 65.7). With regard to rectal cancer, the 64 hospitals were divided into 50 low-volume hospitals conducting a median of 9.5 resections per year (minimum 0.1, maximum 19.7) and 14 high-volume hospitals with a median of 25.4 resections per year (minimum 20.4, maximum 57.0).

**Table 1:** Patient characteristics (2010–2020)

	Colon cancer (C18) n (%)	Rectal cancer (C19/C20) n (%)	Total n (%)
Sex			
Male	8.675 (54.6)	5.311 (64.8)	13.986 (58.1)
Female	7.208 (45.4)	2.891 (35.2)	10.099 (41.9)
**Age (Median)**	74	70	73
**Localization** ^**1**^			
Colon right-sided	9.352 (58.9)	-	9.352 (38.8)
Colon left-sided	6.423 (40.4)	-	6.423 (26.6)
Colon others*	108 (0.7)	-	108 (0.4)
Rectosigmoid junction	-	67 (0.8)	67 (0.3)
Rectum	-	8.135 (99.2)	8.135 (34.0)
**UICC tumor stage**			
I	3.373 (21.2)	1.542 (18.8)	4.915 (20.4)
II	5.611 (35.3)	1.904 (23.2)	7.515 (31.2)
III	4.157 (26.2)	3.612 (44.0)	7.769 (32.3)
IV	2.742 (17.3)	1.144 (13.9)	3.886 (16.1)
**Surgical approach**			
Open	10.194 (64.2)	4.147 (50.6)	14.341 (59.6)
Laparoscopic	2.357 (14.8)	2.033 (24.8)	4.390 (18.2)
Conversion	259 (1.6)	589 (7.2)	848 (3.5)
Others/n. a.	3.073 (19.3)	1.429 (17.4)	4.502 (18.7)
**Urgency of surgery**			
Elective surgery	10.063 (63.4)	5.723 (69.8)	15.786 (65.5)
Emergency surgery	1.198 (7.5)	148 (1.8)	1.346 (5.6)
Unknown/missing	4.622 (29.1)	2.331 (28.4)	6.953 (28.9)
**Number of resected lymph nodes**			
< 12	1.167 (7.5)	1.006 (12.4)	2.173 (9.2)
12+	14.451 (92.5)	7.085 (87.6)	21.536 (90.8)
**Adjuvant chemotherapy**			
no	11.358 (71.5)	4.963 (60.5)	16.321 (67.8)
yes	4.525 (28.5)	3.239 (39.5)	7.764 (32.2)
**Hospital case volume** ^**2**^			
Low	6.965 (43.9)	4.484 (54.7)	11.449 (47.5)
High	8.918 (56.1)	3.718 (45.3)	12.636 (52.5)
**Year of surgery**			
2010–2013	5.374 (33.8)	3.104 (37.8)	8.478 (35.2)
2014–2017	5.819 (36.6)	2.974 (36.3)	8.793 (36.5)
2018–2020	4.690 (29.5)	2.124 (25.9)	6.814 (28.3)
**GISD, Socio-economic deprivation**			
1 - low	3.282 (20.7)	1.584 (19.3)	4.866 (20.2)
2 - mid-low	3.104 (19.5)	1.694 (20.7)	4.798 (19.9)
3 - medium	3.106 (19.6)	1.696 (20.7)	4.802 (19.9)
4 - mid-high	3.624 (22.8)	1.795 (21.9)	5.419 (22.5)
5 - high	2.767 (17.4)	1.433 (17.5)	4.200 (17.4)
**Total number of cases**	**15,883**	**8,202**	**24,085**

^1^ Colon right-sided: C18.0, C18.2-C18.4, Colon left-sided: C18.5-C18.7, Colon others: C18.8-C18.9.

^2^ C18: low < 30 surgeries/year, high ≥ 30 surgeries/year. C19/20: low < 20 surgeries/year, high ≥ 20 surgeries/year.

Abbreviations: GISD, German Index of Socioeconomic Deprivation; UICC, Union for International Cancer Control.

## Survival

### Univariate analysis

**Fig. 3:**
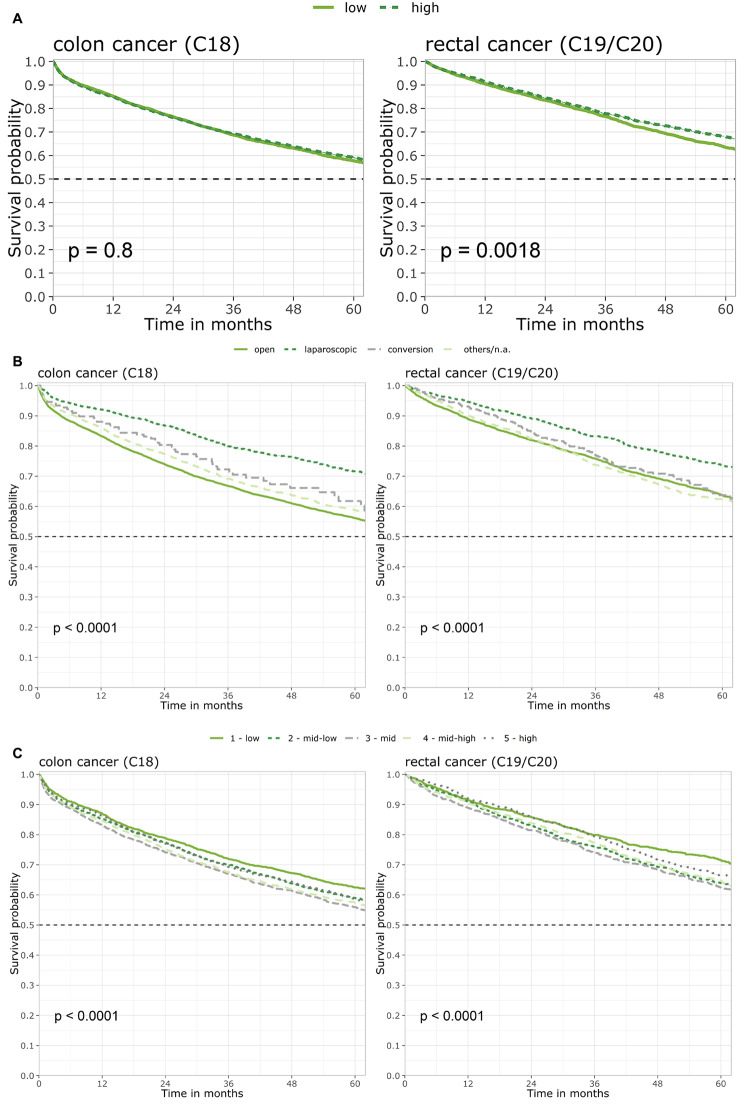
Univariate survival analyses (Kaplan-Meier curves) and results of log-rank test Figure 3A: Univariate survival analysis for surgery in low-volume vs. high-volume hospitals Figure 3B: Univariate survival analysis for surgical approach Figure 3C: Univariate survival analysis for socioeconomic deprivation (GISD-quintiles) Abbreviations: GISD, German Index of Socioeconomic Deprivation.

Median survival time was 87.9 months for colon cancer and 110.0 months for rectal cancer (Table 2). Five years after diagnosis, a total of 58.5% (C18) and 65.6% (C19/C20) of patients were still alive. Males and females had comparable 5-year survival rates (colon: 57.7% vs. 59.5%, *P* = 0.1; rectum: 65.6% vs. 65.6%, *P* = 0.2), whereas patients aged 70 years and younger had better 5-year survival than older patients (colon: 68.8% vs. 51.6%, *P* < 0.001; rectum: 73.7% vs. 55.4%, *P* < 0.001). Survival probability significantly decreased with higher UICC tumor stage (colon: stage I 80.0% vs. stage IV 15.2%; rectum: stage I 80.3% vs. stage IV 26.9%). Further univariate analyses revealed a significant association with surgical approach, hospital case volume (rectum only) and socioeconomic deprivation (Table 2; Fig. 3A–C). Laparoscopic surgery was associated with a better 5-year survival for both colon cancer (71.2% vs. 55.9%, *P* < 0.001) and rectal cancer (73.2% vs. 63.8%, *P* < 0.001). In addition, patients with rectal cancer had better 5-year survival when tumor resection was carried out in a high-volume hospital (68.2% vs. 63.5%, *P* = 0.002). Regarding socioeconomic deprivation, 5-year survival was worse for mid-low, mid and mid-high levels of deprivation compared to a low level of deprivation (overall *P* < 0.001). No difference could be observed for the highest level of deprivation compared to the lowest level of deprivation.

**Table 2:** Results of overall and univariate survival analyses (Kaplan-Meier estimation)

	Colon cancer (C18)		Rectal cancer (C19/C20)	
	Median survival (Months, 95% CI)	5-year survival (%, 95% CI)	Median survival (Months, 95% CI)	5-year survival (%, 95% CI)
**Overall**	87.9 (84.5; 91.6)	58.5 (57.6; 59.5)	110.0 (105.0; 115.0)	65.6 (64.4; 66.8)
**Sex**		*p = 0.1*		*p = 0.2*
Male	84.1 (79.7; 88.9)	57.7 (56.4; 59.0)	105.0 (98.4; 112.0)	65.6 (64.1; 67.2)
Female	92.2 (87.2; 101.1)	59.5 (58.2; 61.0)	121.0 (110.4; n. a.)	65.6 (63.5; 67.7)
**Age**		*p < 0.001*		*p < 0.001*
≤ 70 years	n. a. (134.3.; n. a.)	68.8 (67.4; 70.2)	n. a. (135.9; n. a.)	73.7 (72.2; 75.2)
> 70 years	63.7 (60.9; 67.3)	51.6 (50.4; 52.9)	71.7 (67.2; 76.3)	55.4 (53.5; 57.3)
**UICC tumor stage**		*p < 0.001*		*p < 0.001*
I	134.3 (132.5; n. a.)	80.0 (78.2; 81.8)	n. a. (130.0; n. a.)	80.3 (77.8; 82.8)
II	121.2 (113.1; 131.4)	69.9 (68.4; 71.4)	116.3 (107.6; 128.7)	70.5 (68.1; 73.0)
III	83.2 (76.8; 91.1)	57.9 (56.1; 59.8)	127.7 (115.2; 135.9)	69.6 (67.9; 71.4)
IV	19.3 (17.8; 20.5)	15.2 (13.6; 16.9)	32.5 (30.3; 34.9)	26.9 (24.0; 30.2)
**Surgical approach**		*p < 0.001*		*p < 0.001*
Open	77.4 (73.3; 82.7)	55.9 (54.7; 57.1)	102.1 (94.5; 110.0)	63.8 (62.1; 65.5)
Laparoscopic	138.5 (121.5; n. a.)	71.2 (68.6; 73.9)	129.9 (123.2; n. a.)	73.2 (70.7; 75.8)
Conversion	122.7 (61.8; n. a.)	61.4 (53.3; 70.8)	90.8 (78.8; n. a.)	62.8 (57.7; 68.3)
Others/n. a.	91.4 (85.4; 103.1)	58.4 (56.3; 60.6)	100.5 (84.9; 119.0)	62.1 (59.3; 65.1)
**Hospital case volume** ^**1**^		*p = 0.8*		*p = 0.002*
Low	87.4 (82.2; 94.4)	57.8 (56.3; 59.2)	100.0 (95.2; 112.0)	63.5 (61.8; 65.2)
High	88.0 (84.3; 93.4)	59.2 (57.9; 60.4)	115.0 (109.7; 128.0)	68.2 (66.4; 70.0)
**GISD**		*p < 0.001*		*p < 0.001*
1 – low	104.7 (95.3; 118.3)	62.1 (60.1; 64.2)	129.3 (125.0; n. a.)	71.7 (69.1; 74.4)
2 – mid-low	87.4 (79.7; 93.6)	58.6 (56.5; 60.8)	98.8 (87.8; 114.0)	64.0 (61.3; 66.8)
3 – mid	83.2 (72.4; 91.1)	56.3 (54.1; 58.5)	96.3 (86.9; 105.0)	62.3 (59.6; 65.1)
4 – mid-high	79.9 (74.2; 86.7)	57.0 (55.0; 59.0)	108.6 (99.2; 123.0)	64.4 (61.8; 67.1)
5 - high	90.6 (83.3; 105.3)	58.7 (56.4; 61.1)	120.3 (110.0; n. a.)	66.2 (63.3; 69.2)

^1^ C18: low < 30 surgeries/year, high ≥ 30 surgeries/year. C19/20: low < 20 surgeries/year, high ≥ 20 surgeries/year.

Abbreviation**s**: CI, Confidence interval; GISD, German Index of Socioeconomic Deprivation; n. a., survival probability did not reach 50%; UICC Union for International Cancer Control.

### Multivariate analysis

The results of multivariate analyses, including all relevant variables, are presented in Table 3. Females had better survival rates than males (colon: HR = 0.89, 95% CI = 0.84–0.94, *P <* 0.001; rectum: HR = 0.85, 95% CI = 0.78–0.92, *P <* 0.001) and older age was associated with worse survival (colon: HR = 1.91, 95% CI = 1.79–2.03, *P <* 0.001; rectum: HR = 2.12, 95% CI = 1.95–2.30, *P <* 0.001). Compared to early-stage cancer, increasing UICC was associated with worse survival (colon: HR = 10.81, 95% CI = 9.65–12.11, *P <* 0.001 for stage IV; rectum: HR 7.30, 95% CI = 6.24–8.53, *P <* 0.001). Laparoscopic surgery was associated with better survival compared to open surgery in colon cancer patients (HR = 0.76, 95% CI = 0.68–0.83, *P <* 0.001) as well as rectal cancer patients (HR = 0.87, 95% CI = 0.78–0.98, *P <* 0.01). Survival was worse in case of emergency surgery compared to elective surgery (colon: HR = 1.78, 95% CI = 1.62–1.95, P < 0.001; rectum: HR = 2.14, 95% CI = 1.68–2.74, P < 0.001). A higher number of resected lymph nodes was associated with better survival only in colon cancer patients (HR = 0.78, 95% CI = 0.70–0.87, P < 0.001). Better survival was observed for patients receiving adjuvant chemotherapy (colon: HR = 0.49, 95% CI = 0.46–0.53, P < 0.001; rectum: HR = 0.64, 95% CI = 0.58–0.70, P < 0.001). Patients operated in a high-volume hospital showed better survival for rectal cancer (HR = 0.89, 95% CI = 0.82–0.97, *P <* 0.01), whereas no significant effect was observed for colon cancer patients. Compared to patients living in a low deprivation municipality, those living in areas of mid-low to mid-high deprivation had worse survival outcomes (mid-low: HR = 1.18, 95% CI = 1.08–1.30, *P <* 0.001; mid: HR = 1.18, 95% CI = 1.07–1.29, *P <* 0.001; mid-high: HR = 1.22, 95% CI = 1.12–1.33, *P <* 0.001). Rectal cancer patients showed almost similar results (Table 3).

**Table 3:** Results of multivariate weighted Cox regression

	Colon cancer (C18)		Rectal cancer (C19/C20)	
	Hazard Ratio (95% CI)	p	Hazard Ratio (95% CI)	p
**Sex**				
Male	Reference		Reference	
Female	0.89 (0.84; 0.94)	< 0.001	0.85 (0.78; 0.92)	< 0.001
**Age**				
≤ 70 years	Reference		Reference	
> 70 years	1.91 (1.79; 2.03)	< 0.001	2.12 (1.95; 2.30)	< 0.001
**Localization** ^**1**^				
Colon right-sided	Reference		-	
Colon left-sided	0.92 (0.86; 0.97)	< 0.001	-	
Colon others	1.13 (0.84; 1.53)	0.410	-	
Rectosigmoid junction	-		Reference	
Rectum	-		1.01 (0.61; 1.68)	0.969
**UICC tumor stage**				
I	Reference		Reference	
II	1.51 (1.37; 1.67)	< 0.001	1.60 (1.39; 1.85)	< 0.001
III	2.98 (2.68; 3.31)	< 0.001	1.89 (1.64; 2.16)	< 0.001
IV	10.81 (9.65; 12.11)	< 0.001	7.30 (6.24; 8.53)	< 0.001
**Surgical approach**				
Open	Reference		Reference	
Laparoscopic	0.76 (0.68; 0.83)	< 0.001	0.87 (0.78; 0.98)	< 0.01
Conversion	0.94 (0.73; 1.21)	0.632	1.09 (0.92; 1.28)	0.318
Others/n. a.	0.96 (0.89; 1.04)	0.288	1.05 (0.94; 1.17)	0.383
**Urgency of surgery**				
Elective surgery	Reference		Reference	
Emergency surgery	1.78 (1.62; 1.95)	< 0.001	2.14 (1.68; 2.74)	< 0.001
Unknown/missing	1.06 (0.99; 1.14)	0.887	0.96 (0.88; 1.06)	0.424
**Resected lymph nodes**				
< 12	Reference		Reference	
12+	0.78 (0.70; 0.87)	< 0.001	1.05 (0.93; 1.19)	0.412
**Adjuvant chemotherapy**				
no	Reference		Reference	
yes	0.49 (0.46; 0.53)	< 0.001	0.64 (0.58; 0.70)	< 0.001
**Hospital case volume** ^**2**^				
Low	Reference		Reference	
High	0.97 (0.91; 1.03)	0.303	0.89 (0.82; 0.97)	< 0.01
**Year of surgery**				
2010–2013	Reference		Reference	
2014–2017	0.97 (0.90; 1.03)	0.334	0.89 (0.81; 0.98)	< 0.01
2018–2020	1.06 (0.97; 1.16)	0.233	0.83 (0.72; 0.95)	< 0.001
**GISD**				
1 – low	Reference		Reference	
2 – mid-low	1.18 (1.08; 1.30)	< 0.001	1.36 (1.19; 1.55)	< 0.001
3 – mid	1.18 (1.07; 1.29)	< 0.001	1.34 (1.18; 1.53)	< 0.001
4- mid-high	1.22 (1.12; 1.33)	< 0.001	1.18 (1.03; 1.34)	< 0.01
5 - high	1.06 (0.96; 1.18)	0.240	1.04 (0.90; 1.21)	0.573

^1^ Colon right-sided: C18.0, C18.2-C18.4, Colon left-sided: C18.5-C18.7, Colon others: C18.8-C18.9.

^2^ C18: low < 30 surgeries/year, high ≥ 30 surgeries/year. C19/20: low < 20 surgeries/year, high ≥ 20 surgeries/year.

Abbreviations: CI, Confidence interval; GISD, German Index of Socioeconomic Deprivation; UICC Union for International Cancer Control.

## Conclusion

In accordance with other studies, we confirmed that characteristics like male sex, higher age and increasing UICC tumor stage are risk factors for worse survival [17, 18]. In addition, we found that a laparoscopic approach was associated with better survival. A mid-low, medium and mid-high GISD score was also an independent risk factor for worse survival. Only in rectal cancer patients, treatment in high volume hospitals was associated with better survival.

According to the literature, both the laparoscopic and open techniques seem to be equivalent in terms of tumor-specific survival [19, 20]. However, results from recent trials indicate that an open surgical approach was associated with a higher risk in terms of long-term mortality and OS [21, 22].Our findings also confirmed these data. Nevertheless, the reasons for this remain unclear and speculative. In our data, high-volume hospitals had almost double the percentage of laparoscopic surgeries compared to low-volume hospitals regarding rectal cancer. A more oncological adequate (better mesocolonic/mesorectal excision) resection may have been carried out. Compared with laparoscopic surgery, the open approach can result in postoperative morbidity (e.g., higher rates of incisional hernia, wound infection and other septic complications leading to reduced mortality and OS), which may contribute to the better outcomes following laparoscopic treatment [23]. The presence of a selection bias may also explain the superiority of the laparoscopic technique for overall survival. For elderly patients with higher risk profiles and secondary diseases, some hospitals are more likely to perform open procedures in order to avoid longer anesthesia and operating times. Unfortunately, we could adjust our analyses only for age, not for preexisting diseases or ASA (American Society of Anesthesiologists) risk classification or similar scores, due to missing data.

We are aware that our study has several weaknesses on account of its retrospective character. Due to lack of data (not reported to the clinical cancer registries), no statement about pre-existing illnesses or medication can be made. In Germany, older patients are more often operated in emergency and smaller hospitals,, which could result in a selection bias[24]. In addition, it is not possible to differentiate between high and low rectal cancer due to missing tumor height. No information on robotic surgery or tumor-specific survival is due to the current state of data collection. Use of the GISD is a necessary step because there is no valid better way to assess an individual socioeconomic score by income and educational level. Its limitations are set by the nature of GISD representing the patients’ socioeconomic status using habitation-based data with high individual variability. The fact that the lowest GISD strata did not show worse results is quite interesting. The explanation is speculative, but might occur due to the nature of GISD, which uses population-based and not individual data until 2012, whereas survival and effects of healthcare programs might result in the later episode. Also, you might state, that the quality of medical treatment in areas with highest GISD is quite good.

Only rectal cancer patients profited from treatment in high-volume hospitals achieving better survival. The highest level of evidence provided by the meta-analysis of Huo et al. indicates that the best outcomes occur in high-volume hospitals with high-volume surgeons, followed by low-volume hospitals with high-volume surgeons [9]. Thus, the individual surgeon is the main factor in achieving superior survival and oncological outcome. Unfortunately, the cancer registry data does not provide information on the specific surgeon. Rectal cancer surgery is more complex (narrow space in the pelvis) and less frequent (about one third of all colorectal operations) compared to colon cancer surgery. Training and experience of the surgeon is extremely important to achieve high quality of total mesorectal excision, which is directly associated with better patients’ survival and higher rates of sphincter preserving surgeries [25, 26]. Also, patients profit enormously from a laparoscopic or robotic surgical approach, which needs lots of practice and is more often carried out in high volume hospitals[27–29]. In addition, the fact that only one hospital specializing in rectal surgery had higher numbers than 50 operations per year is quite remarkable, and not competitive to other countries. For a better understanding of these problems in Germany, you need to look at the hospital allocation. In recent years, many hospitals in Germany, including Saxony, introduced evidence-based quality standards and improved their surgical and oncological outcomes [30]. However, since many hospitals are allowed to treat cancer patients, only few hospitals reach high patient volumes. In addition, in each hospital, there is not only one surgeon operating CRC patients, meaning the volume is shared by more surgeons, leading to low individual surgeon case load per year. Consequently, CRC care in Germany remains decentralized with high in-hospital morbidity and mortality rates compared to other western countries like USA, France, and UK [31]. While caseload can serve as a surrogate for treatment quality assessment in CRC surgery, our data do not suggest better survival for high-volume hospitals regarding colon cancer. [30]. Implementing independent controls and an auditing system surely helped maintaining standards [32]. Higher case numbers to achieve an obligatory certification as a pre-condition to treat CRC patients, and financial retribution could lead to more economical impact concerning the treatment of CRC patients [33]. There is a political and structural need for centralization and specialization in order to improve outcomes in the treatment of CRC patients in Saxony, Germany.

Our study also provides further evidence of social disparities in the treatment of CRC. Socioeconomic deprivation in Saxony, Germany is inversely associated with survival in CRC treatment. This association persists after demographic and cancer-related factors are considered. Relatively few data and studies exist on this topic in Germany. For the US, on the other hand, the existence of social and racial discrimination has been clearly demonstrated [34]. These major regional socioeconomic inequalities indicate a high potential for improving cancer care and survival worldwide. As many cancer entities have a long period of latency, changes in lifestyle, nutrition and physical activity, led to changes in incidence over the last decades. As obesity, one of the best-known risk factors for CRC, became epidemic and the nutrition worsened, colorectal cancer incidence increased, especially in rural areas with low income. In comparison to the 1950s, CRC changed from a prosperity disease to a disease of higher prevalence in low-income social stratum [35, 36]. This fact was clearly confirmed by our data indicating rural areas to have higher GISD and thus worse survival. An increased travelling distance to inpatient and outpatient care might be another reason for the high urban- rural disparities. Also, higher education and income, overrepresented in urban areas (lower GISD strata) showed better survival. This could be due to better access to prevention coloscopy and healthier lifestyle. Unfortunately, exact data on this topic is missing. Although, excessive food intake and insufficient physical activity are individual decision, political, economic, and social disparities play a major role in fighting adiposity and CRC[37]. We live in an environment where obesity is stimulated. This might be a key to further decrease CRC incidence and mortality. An efficient allocation of health resources therefore is indispensable.[38].

Our data analysis from Saxony could be extended to the other federal states and Germany as a whole. The data transfer process should also be expanded to create nationwide databases. Therefore, data from health insurance companies could also be added. Since individual data on income, employment status and educational level are often missing, future studies should address individual-level patient data with access to treatment information. This would allow more detailed examination of the reasons for these socioeconomic inequalities in cancer survival and could help establish improvements in access to health protection, diagnostic and therapy[7]. The social disparities leading to an elevated risk for cancer development and worse survival require targeted public health action and policy in order to address the complexity of these relationships [5].

## Summary

In the analyzed population in Saxony, Germany, we showed that laparoscopic surgical technique and lower socioeconomic deprivation were associated with better survival in the treatment of CRC. There was also some indication that high hospital case volume improves patient outcome in rectal cancer. Our study adds evidence that might help in changing national health policies with the aim of achieving a better outcome for patients with CRC. Only a change at the national level can help improve therapy and elevate the quality of colorectal cancer care delivery.

## Electronic supplementary material

Below is the link to the electronic supplementary material.

Supplementary Material 1

## Data Availability

The datasets used and/or analyzed during the current study available from the corresponding author on reasonable request.

## List of Abbreviations

CI: Confidence interval
CRC: Colorectal cancer
GISD: German Index of Socioeconomic Deprivation
HR: Hazard ratio
OS: Overall survival
UICC: Union for international cancer control
