# Supplementary material for: The influence of socioeconomic aspects and hospital case volume on survival in colorectal cancer in Saxony, Germany
Source: BMC Cancer. 2023 Mar 10;23:228. doi: 10.1186/s12885-023-10672-1 (PMC9999591; doi:10.1186/s12885-023-10672-1)
Supplement: Supplementary file 1 — Supplementary Material 1 [file 12885_2023_10672_MOESM1_ESM.docx]

**Supplementary Table 1: Patient characteristics by hospital case volume, colon cancer**

|  | **Low-volume hospitals**  **n (%)** | **High-volume hospitals**  **n (%)** | **p** | **Total**  **n (%)** |
| --- | --- | --- | --- | --- |
| **Sex** |  |  | 0.097 |  |
| Male | 3.752 (53.9) | 4.923 (55.2) |  | 8.675 (54.6) |
| Female | 3.213 (46.1) | 3.995 (44.8) |  | 7.208 (45.4) |
| **Age (Median)** | 74 | 74 | 0.174 | 74 |
| **Localization^1^** |  |  | < 0.001 |  |
| Colon right-sided | 4.011 (57.6) | 5.341 (59.9) |  | 9.352 (58.9) |
| Colon left-sided | 2.884 (41.4) | 3.539 (39.7) |  | 6.423 (40.4) |
| Colon others* | 70 (1.0) | 38 (0.4) |  | 108 0.7 |
| **UICC tumor stage** |  |  | < 0.001 |  |
| I | 1.361 (19.5) | 2.012 (22.6) |  | 3.373 (21.2) |
| II | 2.518 (36.2) | 3.093 (34.7) |  | 5.611 (35.3) |
| III | 1.875 (26.9) | 2.282 (25.6) |  | 4.157 (26.2) |
| IV | 1.211 (17.4) | 1.531 (17.2) |  | 2.742 (17.3) |
| **Surgical approach** |  |  | < 0.001 |  |
| Open | 4.015 (57.6) | 6.179 (69.3) |  | 10.194 (64.2) |
| Laparoscopic | 854 (12.3) | 1.503 (16.9) |  | 2.357 (14.8) |
| Conversion | 110 (1.6) | 149 (1.7) |  | 259 (1.6) |
| Others/n. a. | 1.986 (28.5) | 1.087 (12.2) |  | 3.073 (19.3) |
| **Urgency of surgery** |  |  | < 0.001 |  |
| Elective surgery | 4.384 (62.9) | 5.679 (63.7) |  | 10.063 (63.4) |
| Emergency surgery | 367 (5.3) | 831 (9.3) |  | 1.198 (7.5) |
| Unknown/missing | 2.214 (31.8) | 2.408 (27.0) |  | 4.622 (29.1) |
| **Number of resected lymph nodes** |  |  | < 0.05 |  |
| < 12 | 547 (8.0) | 620 (7.0) |  | 1.167 (7.5) |
| 12+ | 6.259 (92.0) | 8.192 (93.0) |  | 14.451 (92.5) |
| **Adjuvant chemotherapy** |  |  | < 0.001 |  |
| no | 4.850 (69.6) | 6.508 (73.0) |  | 11.358 (71.5) |
| yes | 2.115 (30.4) | 2.410 (27.0) |  | 4.525 (28.5) |
| **Year of surgery** |  |  | 0.742 |  |
| 2010-2013 | 2.390 (34.3) | 2.984 (33.5) |  | 5.374 (33.8) |
| 2014-2017 | 2.540 (36.65) | 3.279 (36.8) |  | 5.819 (36.6) |
| 2018-2020 | 2.035 (29.2) | 2.655 (29.8) |  | 4.690 (29.5) |
| **GISD, Socio-economic deprivation** |  |  | < 0.001 |  |
| 1 - low | 1.161 (16.7) | 2.121 (23.8) |  | 3.282 (20.7) |
| 2 - mid-low | 1.051 (15.1) | 2.053 (23.0) |  | 3.104 (19.5) |
| 3 - medium | 1.864 (26.8) | 1.242 (13.9) |  | 3.106 (19.6) |
| 4 - mid-high | 805 (11.6) | 2.819 (31.6) |  | 3.624 (22.8) |
| 5 - high | 2.084 (29.9) | 683 (7.7) |  | 2.767 (17.4) |
| **Total number of cases** | **6,965** | **8,918** |  | **15,883** |

Low volume < 30 surgeries/year, high volume ≥ 30 surgeries/year.

^1^ Colon right-sided: C18.0, C18.2-C18.4, Colon left-sided: C18.5-C18.7, Colon others: C18.8-C18.9.

**Supplementary Table 2: Patient characteristics by hospital case volume, rectal cancer**

|  | **Low-volume hospitals**  **n (%)** | **High-volume hospitals**  **n (%)** | **p** | **Total**  **n (%)** |
| --- | --- | --- | --- | --- |
| **Sex** |  |  | 0.05 |  |
| Male | 2.860 (63.8) | 2.451 (65.9) |  | 5.311 (64.8) |
| Female | 1.624 (36.2) | 1.267 (34.1) |  | 2.891 (35.2) |
| **Age (Median)** | 71 | 69 | < 0.001 | 70 |
| **Localization^1^** |  |  | < 0.001 |  |
| Rectosigmoid junction | 57 (1.3) | 10 (0.3) |  | 67 (0.8) |
| Rectum | 4.427 (98.7) | 3.708 (99.7) |  | 8.135 (99.2) |
| **UICC tumor stage** |  |  | < 0.001 |  |
| I | 874 (19.5) | 668 (18.0) |  | 1.542 (18.8) |
| II | 1.122 (25.0) | 782 (21.0) |  | 1.904 (23.2) |
| III | 1.898 (42.3) | 1.714 (46.1) |  | 3.612 (44.0) |
| IV | 590 (13.2) | 554 (14.9) |  | 1.144 (13.9) |
| **Surgical approach** |  |  | < 0.001 |  |
| Open | 2.349 (52.4) | 1.798 (48.4) |  | 4.147 (50.6) |
| Laparoscopic | 821 (18.3) | 1.212 (32.6) |  | 2.033 (24.8) |
| Conversion | 241 (5.4) | 348 (9.4) |  | 589 (7.2) |
| Others/n. a. | 1.071 (23.9) | 358 (9.6) |  | 1.429 (17.4) |
| **Urgency of surgery** |  |  | < 0.001 |  |
| Elective surgery | 3.067 (68.4) | 2.656 (71.4) |  | 5.723 (69.8) |
| Emergency surgery | 57 (1.3) | 91 (2.4) |  | 148 (1.8) |
| Unknown/missing | 1.360 (30.3) | 971 (26.1) |  | 2.331 (28.4) |
| **Number of resected lymph nodes** |  |  | < 0.01 |  |
| < 12 | 605 (13.7) | 401 (10.9) |  | 1.006 (12.4) |
| 12+ | 3.796 (86.3) | 3.289 (89.1) |  | 7.085 (87.6) |
| **Adjuvant chemotherapy** |  |  | 0.173 |  |
| no | 2.666 (59.5) | 2.297 (61.8) |  | 4.963 (60.5) |
| yes | 1.818 (40.5) | 1.421 (38.2) |  | 3.239 (39.5) |
| **Year of surgery** |  |  | 0.147 |  |
| 2010-2013 | 1.715 (38.2) | 1.389 (37.4) |  | 3.104 (37.8) |
| 2014-2017 | 1.624 (36.2) | 1.350 (36.3) |  | 2.974 (36.3) |
| 2018-2020 | 1.145 (25.5) | 979 (26.3) |  | 2.124 (25.9) |
| **GISD, Socio-economic deprivation** |  |  | < 0.001 |  |
| 1 - low | 620 (13.8) | 964 (25.9) |  | 1.584 (19.3) |
| 2 - mid-low | 713 (15.9) | 981 (26.4) |  | 1.694 (20.7) |
| 3 - medium | 1.118 (24.9) | 578 (15.5) |  | 1.696 (20.7) |
| 4 - mid-high | 881 (19.6) | 914 (24.6) |  | 1.795 (21.9) |
| 5 - high | 1.157 (25.7) | 281 (7.6) |  | 1.433 (17.5) |
| **Total number of cases** | **4,484** | **3,718** |  | **8,202** |

Low volume < 20 surgeries/year, high volume ≥ 20 surgeries/year.
